# Protection from hydrogen peroxide stress relies mainly on AhpCF and KatA2 in *Stenotrophomonas maltophilia*

**DOI:** 10.1186/s12929-020-00631-4

**Published:** 2020-02-25

**Authors:** Li-Hua Li, Yung-Luen Shih, Jing-Yun Huang, Chao-Jung Wu, Yi-Wei Huang, Hsin-Hui Huang, Yu-Chieh Tsai, Tsuey-Ching Yang

**Affiliations:** 1 Department of Pathology and Laboratory Medicine, Taipei Veterans General Hospital, Taipei, Taiwan; 2 Program of Medical Biotechnology, Taipei Medical University, Taipei, Taiwan; 3 Department of Pathology and Laboratory Medicine, Shin Kong Wu Ho-Su Memorial Hospital, Taipei, Taiwan; 4 School of Medical Laboratory Science and Biotechnology, Taipei Medical University, Taipei, Taiwan; 5 School of Medicine, College of Medicine, Fu-Jen Catholic University, New Taipei City, Taiwan; 6 Department of Laboratory Medicine, Chang-Gung Memorial Hospital, LinKou, Taiwan; 7 Department of Biotechnology and Laboratory Science in Medicine, National Yang-Ming University, Taipei, Taiwan

**Keywords:** *Stenotrophomonas maltophilia*, Catalase, Alkyl hydroperoxidase, Glutathione peroxidase, Hydrogen peroxide stress, OxyR regulator

## Abstract

**Background:**

Aerobically-grown bacteria can be challenged by hydrogen peroxide stress from endogenous aerobic metabolism and exogenously generated reactive oxygen species. Catalase (Kat), alkyl hydroperoxidase (Ahp), and glutathione peroxidase (Gpx) systems are major adaptive responses to H_2_O_2_ stress in bacteria. *Stenotrophomonas maltophilia* is a ubiquitous Gram-negative bacterium equipped with four Kats (KatA1, KatA2, KatMn, and KatE), one Ahp (AhpCF), and three Gpxs (Gpx1, Gpx2, and Gpx3). Here, we systematically investigated how the eight H_2_O_2_ scavenging genes differentially contribute to the low-micromolar levels of H_2_O_2_ generated from aerobic metabolism and high-millimolar levels of H_2_O_2_ from exogenous sources.

**Methods:**

Gene expression was assessed and quantified by reverse transcription-PCR (RT-PCR) and real time quantitative PCR (qRT-PCR), respectively. The contribution of these enzymes to H_2_O_2_ stress was assessed using mutant construction and functional investigation.

**Results:**

Of the eight genes, *katA2*, *ahpCF*, and *gpx3* were intrinsically expressed in response to low-micromolar levels of H_2_O_2_ from aerobic metabolism, and the expression of *katA2* and *ahpCF* was regulated by OxyR. AhpCF and KatA2 were responsible for alleviating aerobic growth-mediated low concentration H_2_O_2_ stress and AhpCF played a critical role for stationary-phase cells. *KatA2* was upregulated to compensate for AhpCF in the case of *ahpCF* inactivation. After exposure to millimolar levels of H_2_O_2_, *katA2* and *ahpCF* were upregulated in an OxyR-dependent manner. KatA2 was the critical enzyme for dealing with high concentration H_2_O_2_. Loss-of-function of KatA2 increased bacterial susceptibility to high concentration H_2_O_2_.

**Conclusions:**

AhpCF and KatA2 are key enzymes protecting *S. maltophilia* from hydrogen peroxide stress.

## Background

In aerobic bacteria, hydrogen peroxide (H_2_O_2_) stress is endogenously generated by aerobic metabolism. Exogenous H_2_O_2_ stress can be generated by chemical processes, competing organisms, and host cells in the environment. Superoxide, H_2_O_2_, and hydroxyl radicals are three main reactive oxygen species (ROS) in aerobic bacteria. Unlike superoxide and hydroxyl radicals, H_2_O_2_ is not a free radical and is less toxic to bacteria. However, distinct from superoxide and hydroxyl radicals, H_2_O_2_ can easily diffuse across cell membranes. Furthermore, hydroxyl radical is the most reactive ROS species and it can be readily generated from H_2_O_2_ in the presence of Fe^2+^ via the Fenton reaction, causing irreversible damage to bacteria [1]. Therefore, effective removal of H_2_O_2_ is critical for bacterial survival.

To prevent H_2_O_2_-mediated damage, aerobic bacterial pathogens must quickly convert H_2_O_2_ into other, less dangerous substances. The most common and efficient systems for bacteria to alleviate H_2_O_2_ stresses are an array of scavenging enzymes [2], including catalase (Kat), glutathione peroxidase (Gpx), and alkyl hydroperoxidase/alkyl hydroperoxide reductase (Ahp) [3]. Catalase directly catalyzes the decomposition of hydrogen peroxide without oxidizing the enzyme itself. Peroxidases detoxify H_2_O_2_ by oxidizing itself and rely on cellular reductants to revive them from the oxidized state. A bacterium can harbor an array of H_2_O_2_ scavenging enzymes, like KatG, KatE, AhpCF, and BtuE in *E. coli* [4], and KatA, KatB, KatC, AhpA, AhpB, AhpCF, and BtuE in *P. aeruginosa* [5]. The H_2_O_2_ scavenging enzymes may differentially function in response to different oxidative stress sources.

OxyR, a LysR family transcription factor, is a well-characterized regulator of the H_2_O_2_ response in Gram-negative bacteria [6]. OxyR contains a regulatory domain and a DNA binding domain. After sensing a H_2_O_2_ threat, OxyR undergoes secondary structure rearrangement by forming a disulfide bond between the two conserved cysteine residues in the regulatory domain, resulting in oxidized OxyR. The oxidized OxyR binds to the promoter region of the target gene via the DNA binding domain, modulating target gene expression as a transcriptional activator or repressor.

*Stenotrophomonas maltophilia* is an aerobic, Gram-negative, γ-proteobacterium that is widely distributed in the soil, water, plant rhizosphere, and hospital equipment [7]. It is also a pathogen that infects cystic fibrosis and immunocompromised patients [8]. Because of its diverse habitats, *S. maltophilia* is expected to be equipped with more effective H_2_O_2_ alleviation systems to adapt to different environmental niches. Analysis of the *S. maltophilia* genome sequence indicates the presence of many H_2_O_2_ scavenging enzymes, including four distinct Kats, three Gpxs, and one alkyl hydroperoxidase/alkyl hydroperoxide reductase system (AhpCF) [9]. Given that three systems contribute to neutralize H_2_O_2_ stresses, a defect in a single system can be compensated by the others. Therefore, a global investigation of the three systems, instead of focusing on one system, is likely to contribute more to our understanding of H_2_O_2_ detoxification in bacteria. To our knowledge, no previous studies have comprehensively investigated the function and interplay among the three antioxidant systems in *S. maltophilia*. This study aimed to provide this information and elucidate the role of these antioxidant enzymes in protecting bacteria against H_2_O_2_ stress from aerobic metabolism or exogenous sources.

## Methods

### Bacterial strains, plasmid, and growth condition

Table S1 lists the bacterial strains, plasmids, and PCR primers used in this study. All primers used in this study were designed based on the genome of *S. maltophilia* K279a.

### Construction of in-frame deletion mutants

The strategy of two-step double cross-over homologous recombination was used for the construction of mutants used in this study. Two PCR amplicons, corresponding to upstream and downstream of the gene intended to delete, were amplified using the paired primer sets and subsequently cloned into pEX18Tc to yield the recombinant plasmids for mutants construction. The primer sets used are KatA1N-F/KatA1N-R and KatA1C-F/KatA1C-R for plasmid pΔKatA1, KatA2N-F/KatA2N-R and KatA2C-F/KatA2C-R for plasmid pΔKatA2, KatMnN-F/KatMnN-R and KatMnC-F/KatMnC-R for plasmid pΔKatMn, KatEN-F/KatEN-R and KatEC-F/KatEC-R for plasmid pΔKatE, AhpCN-F/AhpCN-R and AhpFC-F/AhpFC-R for plasmid pΔAhpCF, Gpx1N-F/Gpx1N-R and Gpx1C-F/Gpx1C-R for plasmid pΔGpx1, Gpx2N-F/Gpx2N-R and Gpx2C-F/Gpx2C-R for plasmid pΔGpx2, and Gpx3N-F/Gpx3N-R and Gpx3C-F/Gpx3C-R for plasmid pΔGpx3 (Table S1). These pEX18Tc-derived plasmids were mobilized into KJ cells by conjugation and the transconjugants selection were performed as descried previously [10]. PCR and DNA sequencing were performed to confirm the correctness of mutants. Double, quadruple, and hepta mutants were constructed from single mutants by the same procedure.

### Construction of complementation plasmids pAhpCF and pKatA2

The 2551-bp PCR amplicon containing intact *ahpCF* genes was obtained by PCR using the primer sets AhpCF-F and AhpCF-R and cloned into pRK415, yielding pAhpCF. An approximate 2.1-kb DNA fragment containing intact *katA2* gene was obtained by PCR using primer sets KatA2N-F and KatA2C-R and cloned into pRK415, generating plasmid pKatA2.

### Dihydrochodamine 123 (DHR123) assay

Overnight cultures were subcultured to fresh LB medium containing 0.9 μg/ml DHR123 with an initial OD_450_ of 0.15. After a 5-h and 24-h incubations, fluorescence was detected using 500 nm as the excitation wavelength and 550 nm as the emission wavelength.

### Reverse transcription-PCR (RT-PCR)

The DNA-free RNA of logarithmic-phase *S. maltophilia* cells was extracted using Total RNA Extraction Kit Mini (ARROWTEC) and reverse transcribed to cDNA by High Capacity cDNA Reverse Transcription Kit (Applied Biosystems). The cDNA of 100 ng was used as template for PCR with the primers indicated. The primer sets used were KatA1Q-F/R for *katA1*, KatA2Q-F/R for *katA2*, KatMnQ-F/R for *katMn*, KatEQ-F/R for *katE*, AhpCQ-F/R for *ahpC*, Gpx1Q-F/R for *gpx1*, Gpx2Q-F/R for *gpx2*, and Gpx3Q-F/R for *gpx3* (Table S1). PCR amplicons were visualized by agarose gel electrophoresis. To check the specificity of primer pairs, control PCRs were performed using the chromosome DNA as the template. Since *smeX* in *S. maltophilia* KJ is intrinsically quiescent [11], it was used as the negative control to assure RNA purity.

### Real time quantitative PCR (qRT-PCR)

The cDNA prepared for the aforementioned RT-PCR assay was used as template for qRT-PCR. qRT-PCR was carried out by the ABI Prism 7000 Sequence Detection System (Applied Biosystems) according to the manufacturer’s protocols. The 16 s rRNA gene was used as an internal control and the transcripts of genes assayed were normalized with the internal control using *ΔΔC*_*T*_ method [12]. Primers used for qRT-PCR were the same as those used for RT-PCR (Table S1). All experiments were performed in triplicate.

### Construction of promoter-*xylE* transcriptional fusion reporter plasmids

Three DNA segments upstream and including the start codons of *gpx3*, *katA2*, and *ahpC* were amplified by PCR using the primer sets of Gpx3N-F/Gpx3N-R, KatA2N-F/KatA2N-R, and AhpCN-F/AhpCN-R, respectively (Table S1). These PCR products were inserted into pRKxylE to place the amplicons upstream of *xylE*, which encodes an enzyme with C23O activity. These plasmids were referred to as pGpx3_xylE_, pKatA2_xylE_, and pAhpC_xylE_, respectively.

### Determination of C23O activity

Catechol 2, 3-dioxygenase (C23O), encoded by a *xylE* gene, catalyzes the hydrolysis of catechol into the yellow 2-hydroxymuconate semialdehyde, which can be quantitatively determined by spectrophotometric analysis at a wavelength of 375 nm. C23O activity were determined spectrophotometrically at 375 nm as described previously [11]. The rate of hydrolysis was calculated by using 44,000 M^− 1^ cm^− 1^ as the extinction coefficient. One unit of enzyme activity (U) was defined as the amount of C23O that converts 1 nmole catechol per min. The C23O specific activity was expressed as U/OD_450nm_.

### Growth kinetic assay

Overnight-cultured strain tested was inoculated into fresh LB medium at the initial OD_450nm_ of 0.15. The OD_450nm_ readings were taken at interval of 3 h for a total time of 24 h.

### H_2_O_2_ susceptibility test (disk diffusion assay)

The strain tested was cultured to mid-log phase and adjusted to a concentration of 10^7^ cells/ml. A 100 μl aliquot was spread evenly over the surface of a LB agar plates. A 10 μl of 20% H_2_O_2_ was spotted onto a sterile paper disk (6 mm in diameter) and the disk was placed on the center of plate. The diameter of growth inhibition zone around disk was measured after a 24-h incubation at 37 °C.

## Results

### Analysis of Kat, AhpC, and Gpx systems in *S. maltophilia* genome

The catalase (Kat), alkyl hydroperoxidase/alkyl hydroperoxide reductase (AhpCF), and glutathione peroxidase (Gpx) systems are three major and extensively reported enzymatic H_2_O_2_ elimination systems in several bacteria. Genome sequence analysis showed that four *kat*, one *ahpCF*, and three *gpx* genes existed in the genome of *S. maltophilia* K279a [9]: Smlt0372 (*katA1*), Smlt1385 (*katA2*), Smlt2537 (*katMn*), Smlt3583 (*katE),* Smlt0841–0840 (*ahpCF*), Smlt3183 (*gpx1*), Smlt3228 (*gpx2*), and Smlt4676 (*gpx3*). In this study, we aimed to assess the roles of the eight enzymes in alleviating hydrogen peroxide stress generated by endogenous aerobic metabolism or by exogenous sources.

### AhpCF and KatA2 contribute to scavenge micromolar H_2_O_2_, and AhpCF play a critical role for stationary-phase cells

**Fig. 1:**
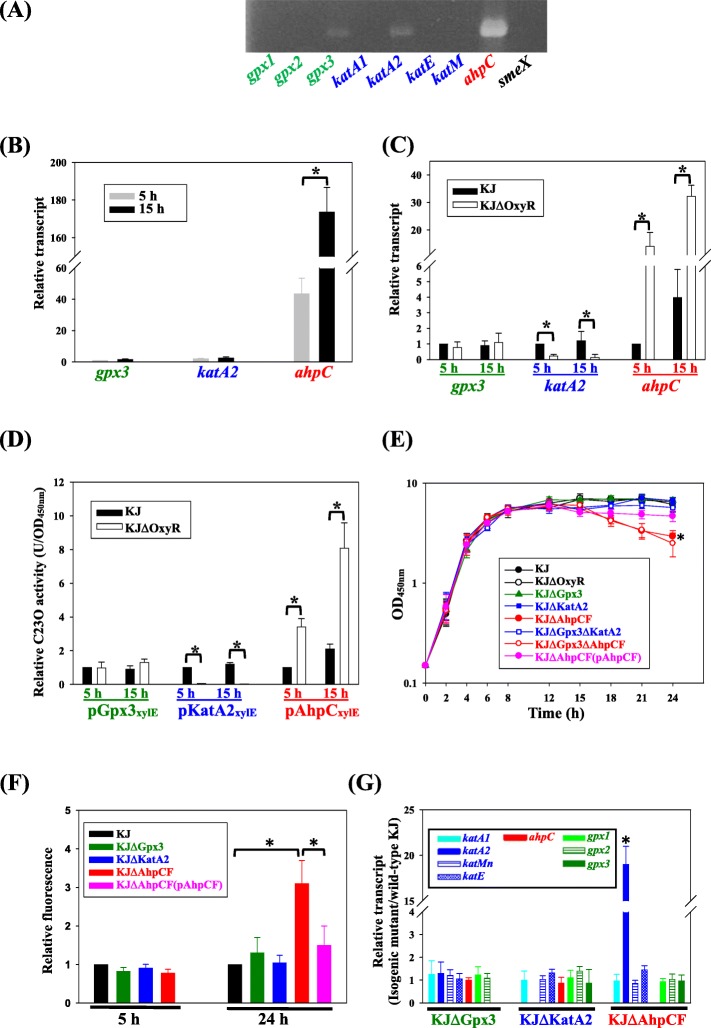
Roles of four catalases (KatA1, KatA2, KatE, and KatMn), one alkyl hydroperoxidase (AhpC), and three glutathione peroxidases (Gpx1, Gpx2, and Gpx3) in the alleviation of endogenous hydrogen peroxide stress. Bars represent the average values from three independent experiments. Error bars represent the standard error of the mean. *, *P* < 0.001, significance calculated by Student’s *t* test. (**a**) Agarose gel electrophoresis of reverse transcription PCR (RT-PCR). Overnight-cultured *S. maltophilia* KJ was inoculated into fresh LB with an initial OD_450nm_ of 0.15 and grown for 5 h. The cDNAs were obtained using reverse transcription with random primers and PCR was performed using primer pairs (Table S1) targeting candidate genes. The *smeX* gene, which is not expressed in strain KJ, is used as a control for DNA contamination during cDNA preparation. (**b**) The expression of *gpx3*, *katA2*, and *ahpC* genes in logarithmic- and stationary-phase wild-type KJ cells. Overnight culture of KJ cells was inoculated into fresh LB with an initial OD_450nm_ of 0.15. Cells were grown aerobically for 5 h or 15 h before measuring *gpx3, katA2,* and *ahpC* transcripts using qRT-PCR. All values were normalized to *gpx3* transcript of logarithmic-phase KJ cells. (**c**) Regulatory role of OxyR in the intrinsic expression levels of *gpx3*, *katA2*, and *ahpC* genes. Overnight cultures of KJ and KJΔOxyR cells were inoculated into fresh LB with an initial OD_450nm_ of 0.15. Cells were grown aerobically for 5 h or 15 h before measuring *gpx3, katA2,* and *ahpC* transcripts using qRT-PCR. All values were normalized to the transcript of logarithmic-phase KJ cells. (**d**) Regulatory role of OxyR in the intrinsic expression levels of *gpx3*, *katA2*, and *ahpC* genes. Overnight cultures of bacteria cells (KJ (Gpx3_xylE_), KJ (pKatA2_xylE_), KJ (pAhpC_xylE_), KJΔOxyR (Gpx3_xylE_), KJΔOxyR (pKatA2_xylE_), and KJΔOxyR (pAhpC_xylE_)) were inoculated into fresh LB with an initial OD_450nm_ of 0.15. Cells were grown aerobically for 5 h or 15 h before measuring the C23O activity. All values were normalized to the activity in KJ cells. (**e**) Functions of OxyR, Gpx, Kat, and AhpCF systems in response to endogenously aerobic metabolism-derived H_2_O_2_ stress. The growth curves of KJ and its derived isogenic mutants were measured by reading OD_450_ at the time points as indicated. *, the growth difference of KJΔAhpCF and KJΔAhpCF (pAhpCF) at the 24-h time point was significant. (**f**) DHR 123 assay of wild-type KJ and mutants KJΔGpx3, KJΔKatA2, and KJΔAhpCF. The bacterial cells tested were cultured in LB medium containing DHR 123 for 5 h and 24 h, respectively, and the fluorescence at 550 nm was determined. The relative fluorescence is normalized to the fluorescence of wild-type KJ. (**g**) The expression levels of *gpxs*, *kats*, and *ahpCF* of KJΔGpx3, KJΔKatA2, and KJΔAhpCF in response to endogenously aerobic metabolism-derived H_2_O_2_ stress. Bacteria cultured overnight (KJ, KJΔGpx3, KJΔKatA2, and KJΔAhpC) were inoculated into fresh LB with an initial OD_450nm_ of 0.15 and grown for 5 h. The *katA1, katA2, katMn, katE, ahpC, gpx1, gpx2, and gpx3* transcripts were measured using qRT-PCR. The relative transcription level for each gene was expressed as the ratio of mutant to wild-type

The intrinsic expression of the H_2_O_2_ scavenging enzyme genes was tested using reverse transcription-PCR (RT-PCR). Of the eight genes tested, *gpx3*, *katA2,* and *ahpC* transcripts were detected (Fig. 1a), suggesting that Gpx3, KatA2, and AhpCF may participate in the alleviation of endogenous H_2_O_2_ stress arising from bacterial aerobic metabolism. The expressions of *gpx3*, *katA2*, and *ahpC* genes in the logarithmic and stationary phases were further assessed by qRT-PCR. The *ahpC* expression was abundant compared to *katA2* and *gpx3* in logarithmic phase. The expression level of *ahpC* was further increased in the stationary phase (Fig. 1b). These observations suggested a critical role for *ahpC* in endogenous H_2_O_2_ stress alleviation.

OxyR is a well-known regulator response to H_2_O_2_ stress in several bacteria [13]. The regulatory role of OxyR in the intrinsic expression of *gpx3*, *katA2* and *ahpC* was assessed by qRT-PCR. The expression of *gpx3* was little affected by OxyR. The *katA2* transcript was obviously decreased in the *oxyR* null mutant, indicating that OxyR is a positive regulator for the intrinsic expression of *katA2*. Nevertheless, OxyR acted as a repressor for the expression of *ahpC* in aerobically-grown cells (Fig. 1c). This observation is peculiar since OxyR is a positive regulator of antioxidant system widely reported in several bacteria [13, 14]; thus we used promoter-*xylE* transcriptional fusion construct to recheck the role of OxyR in the expression of *gpx3*, *katA2*, and *ahpC*. The same conclusion was obtained from the results of promoter-*xylE* transcriptional fusion assay (Fig. 1d). To investigate the roles of *gpx3*, *katA2*, and *ahpCF* in the alleviation of endogenously aerobic metabolism-derived H_2_O_2_ stress, we investigated the aerobic growth of different single mutants (KJΔGpx3, KJΔKatA2, and KJΔAhpCF) and different combinations of double mutants (KJΔGpx3ΔKatA2 and KJΔGpx3ΔAhpCF). After several tries, we could not successfully obtain the double mutant of *katA2* and *aphCF* genes. In addition, KJΔOxyR was also included. None of the tested mutants showed any observable growth restrictions in the logarithmic phase. However, *ahpCF*-associated mutants (KJΔAhpCF and KJΔGpx3ΔAhpCF) exhibited gradual reduction of cell density in the stationary phase, and this compromise was not observed when *ahpCF* genes were complemented (Fig. 1e).

To assess the relatedness of deletion mutant phenotypes to the intracellular H_2_O_2_ concentrations, the intracellular H_2_O_2_ concentrations of wild-type KJ and mutants KJΔGpx3, KJΔKatA2, and KJΔAhpCF in the logarithmic (5 h) and the stationary phases (24 h) were determined by dihydrochodamine 123 (DHR123) assay. DHR123 is used for the detection of intracellular ROS and can detect H_2_O_2_ in the presence of endogenous peroxidases. The presence of ROS oxidizes DHR123 to the fluorescent derivative rhodamine 123. Thus, the intracellular H_2_O_2_ concentration is proportional to the fluorescence intensity. The fluorescences detected from the logarithmic-phase KJΔGpx3, KJΔKatA2, and KJΔAhpCF, and from the stationary-phase KJΔGpx3 and KJΔKatA2 were comparable to that from wild-type KJ (Fig. 1f). Nevertheless, stationary-phase KJΔAhpCF cells had higher fluorescence relative to stationary-phase KJ cells (Fig. 1f), correlated well with stationary-phase growth compromise of *ahpCF-*associated mutants (Fig. 1e).

Given functional redundancy in these H_2_O_2_-alleviating enzymes, we considered the possibility that some of these enzymes may be induced to compensate for the absence of one. To test this hypothesis, the transcription levels of the eight genes were measured using qRT-PCR in the deletion mutants KJΔGpx3, KJΔKatA2, and KJΔAhpCF. Inactivation of *gpx3* or *katA2* alone did not significantly affect the expression of the other seven genes. However, the expression of *katA2* in KJΔAhpCF cells increased by 19 ± 2-fold compared to parental KJ cells (Fig. 1g).

### KatA2 and AhpCF, mainly KatA2, contribute to scavenge millimolar H_2_O_2_

**Fig. 2:**
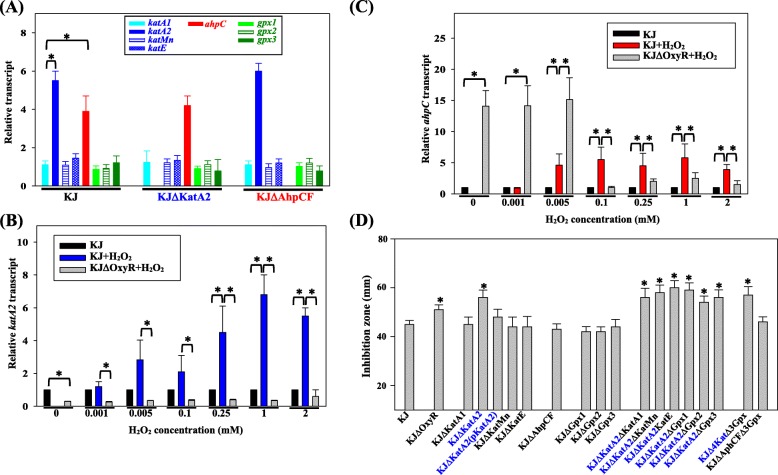
Roles of four catalases (KatA1, KatA2, KatE, and KatMn), one alkyl hydroperoxidase (AhpC), and three glutathione peroxidases (Gpx1, Gpx2, and Gpx3) in the alleviation of exogenous hydrogen peroxide stress. Bars represent the average values from three independent experiments. Error bars represent the standard error of the mean. *, *P* < 0.001, significance calculated by Student’s *t* test. (**a**) Expression of H_2_O_2_-hydrolyzing enzyme genes in the strains KJ, KJΔKatA2, and KJΔAhpC after hydrogen peroxide challenge. The bacteria tested were treated with 2 mM H_2_O_2_ for 10 min before measuring *katA1, katA2, katMn, katE, ahpC, gpx1, gpx2, and gpx3* transcription using qRT-PCR. All values were normalized to individual transcripts obtained from untreated KJ cells. (**b**) Regulatory role of OxyR in *katA2* expression in response to exogenous H_2_O_2_ stress. The KJ and KJΔOxyR cells were untreated or treated with different H_2_O_2_ concentration as indicated for 10 min before measuring *katA2* transcript using qRT-PCR. All values were normalized to *katA2* transcript obtained from untreated KJ cells. (**c**) Regulatory role of OxyR in *ahpC* expression in response to exogenous H_2_O_2_ stress. The KJ and KJΔOxyR cells were untreated or treated with different H_2_O_2_ concentration as indicated for 10 min before measuring *ahpC* transcript using qRT-PCR. All values were normalized to *ahpC* transcript obtained from untreated KJ cells. (**d**) H_2_O_2_ susceptibility test of KJ and its derived isogenic mutants. The bacterial cell suspension tested was uniformly spread onto MH agar, and a sterile filter paper with 10 μl of 20% H_2_O_2_ was placed on the agar. After a 24-h incubation at 37 °C, the growth inhibition zone was measured

The impact of exogenous H_2_O_2_ stress on the expression of H_2_O_2_ scavenging enzymes was investigated by qRT-PCR. Of the eight genes assessed, *katA2* and *ahpC* were upregulated after a 2 mM H_2_O_2_ challenge (Fig. 2a)_._

We also assessed the possibility of compensatory expression in KJΔKatA2 and KJΔAhpCF in the presence of exogenous H_2_O_2_ stress. In either KJΔKatA2 or KJΔAhpCF, the expression levels of the remaining seven H_2_O_2_ scavenging enzymes in response to H_2_O_2_ challenge were hardly affected compared to that in wild-type KJ (Fig. 2a).

We investigated the regulatory role of OxyR in exogenous H_2_O_2_-mediated *katA2* and *ahpC* upregulation with the H_2_O_2_ concentrations ranged from 0 to 2 mM. When the exogenous H_2_O_2_ concentration was as low as 1 μM, there was no impact on the amounts of *katA2* and *ahpC* transcripts. In response to 5 μM or 100 μM H_2_O_2_ challenge, *katA2* transcript had a mild (approximately 2–3 fold), but not significant increment; however, *ahpC* transcript was upregulated (Fig. 2b & c). When the challenged H_2_O_2_ concentration was higher than 250 μM, the *katA2* and *ahpC* transcripts were significantly increased (Fig. 2b & c). In addition, *katA2* expression was positively regulated by OxyR without or with the treatment of H_2_O_2_ (Fig. 2b). However, OxyR regulatory role in *ahpC* expression was H_2_O_2_ concentration dependent, as a repressor when H_2_O_2_ concentration was less than 5 μM and as an activator when H_2_O_2_ concentration was higher than 100 μM (Fig. 2c).

To investigate the role of the eight enzymes in exogenous H_2_O_2_ detoxification, we performed an H_2_O_2_ susceptibility test of KJ-derived mutants containing single deletions of the *katA1*, *katA2*, *katMn*, *katE*, *ahpCF*, *gpx1*, *gpx2*, and *gpx3* genes. In addition, we assessed the H_2_O_2_ susceptibility of KJΔOxyR. Except for KJΔKatA2 and KJΔOxyR, the remaining seven mutants displayed H_2_O_2_ susceptibility that was similar to wild-type KJ (Fig. 2d). KJΔKatA2 was more sensitive to H_2_O_2_ than wild-type KJ (Fig. 2d), and complementation of the mutant with pKatA2, a plasmid containing an intact *katA2* gene, restored H_2_O_2_ resistance (Fig. 2d). KJΔOxyR was also more sensitive to H_2_O_2_ than wild-type KJ, but not as severe as KJΔKatA2 (Fig. 2d). Next, we assessed whether additional mutations in KJΔKatA2 could enhance H_2_O_2_ sensitivity by constructing several combinations of multiple genes deletion mutants using KJΔKatA2 as a parental strain and performing H_2_O_2_ sensitivity assays in all mutants. H_2_O_2_ sensitivity was hardly augmented compared to KJΔKatA2 in all mutants tested, although 4 catalase genes and three *gpx* genes were simultaneously inactivated (KJΔ4KatΔ3Gpx) (Fig. 2d).

It has been reported that OxyR of *E. coli* binds to the 5′ promoter-operator regions of target genes at a conserved motif comprised of four ATAG elements spaced at 10-bp intervals [15, 16]. Since OxyR is involved in the H_2_O_2_-induced upregulation of *katA2* and *ahpCF*, we surveyed the upstream region of the *ahpCF* and *katA2* genes. We discovered ATAG-N14-ATAG and ATAG-N19-ATAG elements near the *ahpCF* and *katA2* promoters (Fig. S1).

## Discussion

H_2_O_2_ stress is an inevitable challenge for aerobic bacteria. Respiratory bursts account for up to 87% of the total H_2_O_2_ production in aerobically-grown *Escherichia coli,* and intracellular H_2_O_2_ from aerobic metabolism normally remains at low-micromolar ranges (< 4 μM) [17]. In the course of infection, H_2_O_2_ levels can reach up to millimolar concentrations because of the oxidative burst generated by host immune cells [2]. To avoid H_2_O_2_ toxicity, bacteria have equipped themselves with several scavenging enzymes to maintain intracellular H_2_O_2_ at nanomolar concentrations [4, 17]. AhpCF and catalase systems are scavenging enzymes that are extensively conserved in several bacterial lineages [2]. AhpCF is more kinetically efficient than catalases at scavenging H_2_O_2_, but its activity is more easily saturated than that of catalases [4]. Therefore, AhpCF is the primary scavenger when H_2_O_2_ is in the low-micromolar range, and catalase activity predominates when the cell reaches millimolar levels of H_2_O_2_ [4]. This paradigm has been observed in a variety of organisms [4], and we highlight our findings in this study to add new evidence to this paradigm.

AhpCF of *S. maltophilia* was expressed in the logarithmic phase and further upregulated in the stationary phase (Fig. 1b), implying that higher AhpCF activity is required for *S. maltophilia* to deal with H_2_O_2_ stress in the stationary phase. This inference is supported by the observation in Fig. 1e and Fig. 1f, since *ahpCF-*associated mutants (KJΔAhpCF and KJΔGpx3ΔAhpCF) exhibited compromised stationary-phase growth (Fig. 1e) and the higher H_2_O_2_ concentration was observed in the stationary-phase KJΔAhpCF cells (Fig. 1f). Inactivation of *katA2* did not affect the expression of other H_2_O_2_ scavenging enzymes (Fig. 1g) and did not compromise bacterial aerobic growth (Fig. 1e), indicating that AhpCF alone is potent enough to deal with the low-micromolar H_2_O_2_ stress. In contrast, upregulation of KatA2 is needed to attain normal logarithmic growth in the case of *ahpCF* inactivation (KJΔAhpCF) (Fig. 1e and g). Collectively, for an aerobically-grown *S. maltophilia*, AhpCF and KatA2 are key enzymes responsible for the alleviation of logarithmic-phase H_2_O_2_ stress and AhpCF system plays a critical role in dealing with the stationary-phase H_2_O_2_ stress.

When bacteria encounter exogenous H_2_O_2_ stress up to the high-micromolar, even millimolar level, *ahpCF* and *katA2* are upregulated (Fig. 2a), linking the contribution of AhpCF and KatA2 to alleviate high concentration H_2_O_2_. However, neither KJΔKatA2 nor KJΔAhpCF exhibited compensatory expression of other enzymes tested in response to 2 mM H_2_O_2_ challenge (Fig. 2a), suggesting that there should be other non-enzymatic systems contributing to deal with millimolar H_2_O_2_ stress in addition to KatA2 and AhpCF. However, we also observed that the *katA2*-associated mutants, but not the other mutants, had a compromised H_2_O_2_ tolerance (Fig. 2d), indicating that among the enzymes tested in this study, KatA2 is the dominant enzyme for the alleviation of high concentration H_2_O_2_ stress.

Vattanaviboon’s group has investigated the role of AhpCF of *S. maltophilia* in response to high level of H_2_O_2_ stress recently [18], and their conclusions are not totally consistent with our findings. They demonstrated that inactivation of *ahpC* rendered *S. maltophilia* more resistant to 300–900 mM H_2_O_2_ than parental strain, which was attributed to the enhanced KatA2 expression and activity [18]. However, our results showed that the expression of *katA2* in the 2 mM H_2_O_2_-treated *ahpCF* mutant (KJΔAhpCF) was comparable to that of parental strain (KJ) (Fig. 2a). The discrepancy may be attributed to different stress intensities (the treated H_2_O_2_ concentration and time intervals), different experiment designs for H_2_O_2_ tolerance evaluation, and strain variation. If the *ahpC* mutant indeed gains a survival superiority against H_2_O_2_ at concentrations commonly used in a hospital, the prevalence of *ahpC* mutant in the clinical *S. maltophilia* isolates should be an interesting issue to study.

**Fig. 3:**
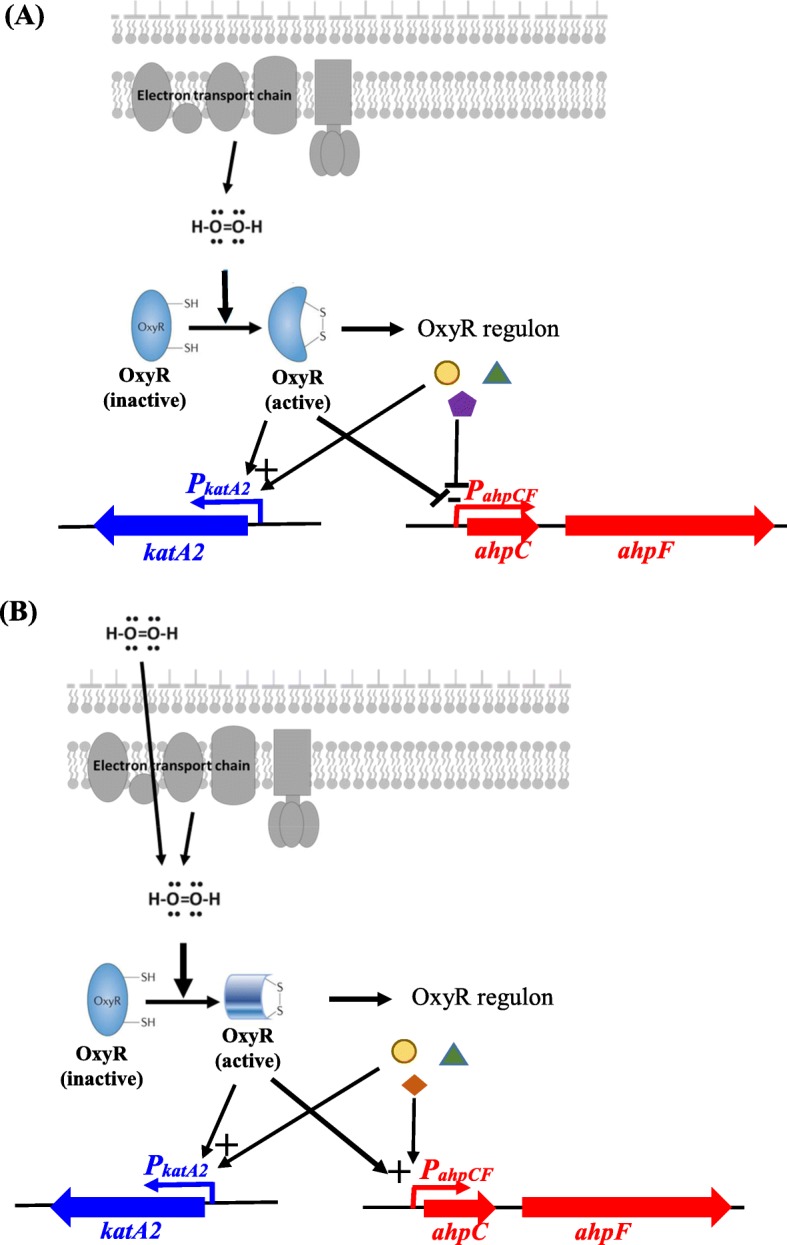
A model for H_2_O_2_-dependent and OxyR-mediated transcription regulation of *ahpCF* and *katA2* genes in response to different concentrations of H_2_O_2_ stress in *S. maltophilia*. (**a**) Low-micromolar H_2_O_2_ is generated by bacterial aerobic metabolism and OxyR is oxidized at a specific “sensing” cysteine residue by H_2_O_2_. The activated OxyR represses the expression of *ahpCF* operon and increases the expression of *katA2* gene, either directly or indirectly. (**b**) When bacteria encounter exogenous H_2_O_2_ stress and the intracellular H_2_O_2_ concentration increases to millimolar levels, activated OxyR activates the expression of *ahpCF* operon and *katA2* gene, either directly or indirectly

The OxyR regulatory role is another interesting finding in this study. OxyR is an H_2_O_2_-sensing transcriptional regulator that is generally conserved in Gram-negative bacteria [13, 14]. In this study, H_2_O_2_ dose-dependent regulation was observed in *S. maltophilia* OxyR. OxyR functioned as a positive regulator for the expression of *katA2* either at micromolar or at millimolar H_2_O_2_ concentrations (Fig. 1c, d, & b). However, OxyR played a double-edged role in the regulation of *ahpCF* expression. OxyR repressed *ahpCF* expression at low-micromolar H_2_O_2_ concentrations (H_2_O_2_ concentration < 5 μM) (Fig. 1c, d & c), but activated *ahpCF* expression when H_2_O_2_ H_2_O_2_ concentration higher than 100 μM (Fig. 2c). This is uncommon because OxyR generally promotes *ahpCF* expression in other bacteria [19]. Herein, we proposed two possibilities to explain this observation. (i) Two different OxyR activated forms may form dependent on the H_2_O_2_ concentrations (different symbols for active OxyR in Fig. 3a and b), which may have different impacts on *ahpCF* expression (Fig. 3). (ii) Members of OxyR regulon triggered by low H_2_O_2_ concentrations are not totally the same as those triggered by high H_2_O_2_ concentrations, and different OxyR regulon member(s) regulate(s) the *ahpCF* expression in micromolar and millimolar H_2_O_2_ concentrations, respectively (Fig. 3a and b). The negative regulatory role of OxyR in *ahpCF* expression (Fig. 1c, d) may help *S. maltophilia* to cope with the endogenous H_2_O_2_ stress in the case of the loss of OxyR function. When *oxyR* is inactivated, the shortage of KatA2 activity can be compensated by upregulated AhpCF, which can maintain normal H_2_O_2_ detoxification. This may be the reason why KJΔOxyR displayed comparable growth with wild-type KJ, but KJΔAhpCF had a growth compromise in the stationary phase (Fig. 1e).

## Conclusion

AhpCF and KatA2 are two main enzymes to differentially protect *S. maltophilia* from the hydrogen peroxide stress. AhpCF and KatA2 participate the alleviation of low-micromolar level H_2_O_2_, and AhpCF has a crucial role for stationary-phase cells; in contrast, KatA2 is the major contributor for dealing with the millimolar level H_2_O_2_. OxyR acts as a positive regulator for the expression of *katA2*. However, the regulatory role of OxyR in the *ahpCF* expression depends on the H_2_O_2_ concentration, as a repressor in H_2_O_2_ of low-micromolar level and as an activator in H_2_O_2_ of millimolar level.

## Supplementary information

**Additional file 1 Table S1.** Bacterial strains, plasmids and primers used in this study.

**Additional file 2 Fig. S1.** Analysis of putative OxyR binding motifs in the upstream regions of *katA2* and *ahpC* genes.

## Data Availability

Data and materials related to this study are available upon request.

## Abbreviations

Ahp: Alkyl hydroperoxidase
DHR123: Dihydrochodamine 123
Gpx: Glutathione peroxidase
Kat: Catalase
QRT-PCR: Real time quantitative PCR
ROS: Reactive oxygen species
RT-PCR: Reverse transcription-PCR

## References

[1] Cabiscol E, Tamarit J, Ros J (2000). Oxidative stress in bacteria and protein damage by reactive oxygen species. Int Microbiol.

[2] Mishra S, Imlay J (2012). Why do bacteria use so many enzymes to scavenge hydrogen peroxide?. Arch Biochem Biophys.

[3] Poole LB (2005). Bacterial defenses against oxidants: mechanistic features of cysteine-based peroxidases and their flavoprotein reductases. Arch Biochem Biophys.

[4] Seaver LC, Imlay JA (2001). Alkyl hydroperoxide reductase is the primary scavenger of endogenous hydrogen peroxide in *Escherichia coli*. J Bacteriol.

[5] Ochsner UA, Vasil ML, Alsabbagh E, Parvatiyar K, Hassett DJ (2000). Role of the *Pseudomonas aeruginosa oxyR-recG* operon in oxidative stress defense and DNA repair: OxyR-dependent regulation of *katB-ankB*, *ahpB*, and *ahpC-ahpF*. J Bacteriol.

[6] Demple B (1991). Regulation of bacterial oxidative stress genes. Annu Rev Genet.

[7] Alavi P, Starcher MR, Thallinger GG, Zachow C, Muller H, Berg G (2014). *Stenotrophomonas* comparative genomics reveals genes and functions that differentiate beneficial and pathogenic bacteria. BMC Genomics.

[8] Parkins MD, Floto RA (2015). Emerging bacterial pathogens and changing concepts of bacterial pathogenesis in cystic fibrosis. J Cyst Fibros.

[9] Crossman LC, Gould VC, Dow JM, Vernikos GS, Okazaki A, Sebaihia M (2008). The complete genome, comparative and functional analysis of *Stenotrophomonas maltophilia* reveals an organism heavily shielded by drug resistance determinants. Gen Biol.

[10] Yang TC, Huang YW, Hu RM, Huang SC, Lin YT (2009). AmpD_I_ is involved in expression of the chromosomal L1 and L2 β-lactamases of *Stenotrophomonas maltophilia*. Antimicrob Agents Chemother.

[11] Chen CH, Huang CC, Chung TC, Hu RM, Huang YW, Yang TC (2011). Contribution of resistance-nodulation-division efflux pump operon *smeU1-V-W-U2-X* to multidrug resistance of *Stenotrophomonas maltophilia*. Antimicrob Agents Chemother.

[12] Livak KJ, Schmittgen TD (2001). Analysis of relative gene expression data using real-time quantitative PCR and the 2-(Delta Delta C(T)) method. Methods.

[13] Dubbs JM, Mongkolsuk S (2012). Peroxide-sensing transcriptional regulators in bacteria. J Bacteriol.

[14] Chen H, Xu G, Zhao Y, Tian B, Lu H, Yu X, Xu Z, Ying N, Hu S, Hua Y (2008). A novel OxyR sensor and regulator of hydrogen peroxide stress with one cysteine residue in *Deinococcus radiodurans*. PLoS One.

[15] Tartaglia LA, Gimeno CJ, Storz G, Ames BN (1992). Multidegenerate DNA recognition by the OxyR transcriptional regulator. J Biol Chem.

[16] Toledano MB, Kullik I, Trinh F, Baird PT, Schneider TD, Storz G (1994). Redox-dependent shift of OxyR-DNA contacts along an extended DNA-binding site: a mechanism for differential promoter selection. Cell.

[17] González-Flecha B, Demple B (1995). Metabolic sources of hydrogen peroxide in aerobically growing *Escherichia coli*. J Biol Chem.

[18] Charoenlap N, Jiramonai L, Chittrakanwong J, Tunsakul N, Mongkolsuk S, Vattanaviboon P (2019). Inactivation of *ahpC* renders *Stenotrophomonas maltophilia* resistant to the disinfectant hydrogen peroxide. Antonie Van Leeuwenhoek.

[19] Hassett DJ, Alsabbagh E, Parvatiyar K, Howell ML, Wilmott RW, Ochsner UA (2000). A protease-resistant catalase, KatA, released upon cell lysis during stationary phase is essential for aerobic survival of a *Pseudomonas aeruginosa oxyR* mutant at low cell densities. J Bacteriol.

