# Supplementary material for: Protection from hydrogen peroxide stress relies mainly on AhpCF and KatA2 in Stenotrophomonas maltophilia
Source: J Biomed Sci. 2020 Feb 25;27:37. doi: 10.1186/s12929-020-00631-4 (PMC7041247; doi:10.1186/s12929-020-00631-4)
Supplement: Supplementary file 1 — Additional file 1 Table S1. Bacterial strains, plasmids and primers used in this study. [file 12929_2020_631_MOESM1_ESM.docx]

**TABLE S1. Bacterial strains, plasmids and primers used in this study**

| **Strain, plasmid, or primer** | **Genotype or properties** | **Reference** |
| --- | --- | --- |
| ***S. maltophilia*** |  |  |
| KJ  KJΔOxyR  KJΔKatA1  KJΔKatA2  KJΔKatMn  KJΔKatE  KJΔAhpCF  KJΔGpx1  KJΔGpx2  KJΔGpx2  KJΔGpx3ΔKatA2  KJΔGpx3ΔApCF  KJΔKtA2ΔKtA1  KJΔKatA2ΔKatMn  KJΔKatA2ΔKatE  KJΔKatA2ΔGpx1  KJΔKatA2ΔGpx2  KJΔKatA2ΔGpx3  KJΔ4KatΔ3Gpx  KJΔAhpCFΔ3Gpx | Wild type, a clinical isolate from Taiwan  *S. maltophilia* KJ *oxyR* mutant; *ΔoxyR*  *S. maltophilia* KJ *katA1* mutant; *ΔkatA1*  *S. maltophilia* KJ *katA2* mutant; *ΔkatA2*  *S. maltophilia* KJ *katMn* mutant; *ΔkatMn*  *S. maltophilia* KJ *katE* mutant; *ΔkatE*  *S. maltophilia* KJ *ahpCF* mutant; *ΔahpCF*  *S. maltophilia* KJ *gpx1* mutant; *Δgpx1*  *S. maltophilia* KJ *gpx2* mutant; *Δgpx1*  *S. maltophilia* KJ *gpx3* mutant; *Δgpx1*  *S. maltophilia* KJ *gpx3* and *katA2* double mutant; *Δgpx3, ΔkatA2*  *S. maltophilia* KJ *gpx3* and *ahpCF* double mutant; *Δgpx3, ΔahpCF*  *S. maltophilia* KJ *katA2* and *katA1* double mutant; *ΔkatA2, ΔkatA1*  *S. maltophilia* KJ *katA2* and *katMn* double mutant; *ΔkatA2, ΔkatMn*  *S. maltophilia* KJ *katA2* and *katE* double mutant; *ΔkatA2, ΔkatE*  *S. maltophilia* KJ *katA2* and *gpx1* double mutant; *ΔkatA2, Δgpx1*  *S. maltophilia* KJ *katA2* and *gpx2* double mutant; *ΔkatA2, Δgpx2*  *S. maltophilia* KJ *katA2* and *gpx3* double mutant; *ΔkatA2, Δgpx3*  *S. maltophilia* KJ *katA1, katA2, katMn, katE, gpx1, gpx2,* and *gpx3* hepta mutant; *ΔkatA1, ΔkatA2, ΔkatMn, ΔkatE, Δgpx1, Δgpx2, Δgpx3*  *S. maltophilia* KJ *ahpCF, gpx1, gpx2,* and *gpx3* quadruple mutant; *ΔahpCF, Δgpx1, Δgpx2, Δgpx3* | 1  2  This study  This study  This study  This study  This study  This study  This study  This study  This study  This study  This study  This study  This study  This study  This study  This study  This study  This study |
| *Escherichia coli* |  |  |
| DH5α | F- φ80d*lacZ*Δ*M15* Δ(*lacZYA-argF*)*U169 deoR recA1 endA1* *hsdR17* (r_k_^-^ m_k_^+^) *phoA supE44λ^-^* *thi-1 gyrA96 relA1* | Invitrogen |
| S17-1  **Plasmids** | λ pir + mating strain | 3 |
| pEX18Tc | *sacB oriT*, Tc^r^ | 4 |
| pRK415 | Broad host range expression vector, Tc^r^ | 5 |
| pΔKatA1 | pEX18Tc with an internal deletion *katA1* gene; Tc^r^ | This study |
| pΔKatA2 | pEX18Tc with an internal deletion *katA2* gene; Tc^r^ | This study |
| pΔKatMn | pEX18Tc with an internal deletion *katMn* gene; Tc^r^ | This study |
| pΔKatE | pEX18Tc with an internal deletion *katE* gene; Tc^r^ | This study |
| pΔAhpCF | pEX18Tc with an internal deletion *ahpCF* gene; Tc^r^ | This study |
| pΔGpx1 | pEX18Tc with an internal deletion *gpx1* gene; Tc^r^ | This study |
| pΔGpx2 | pEX18Tc with an internal deletion *gpx2* gene; Tc^r^ | This study |
| pΔGpx3 | pEX18Tc with an internal deletion *gpx3* gene; Tc^r^ | This study |
| pKatA2 | pRK415 with an intact *katA2* gene; Tc^r^ | This study |
| pAhpCF  pGpx3_xylE_  pKatA2_xylE_  pAhpC_xylE_  **Primers**  KatA1N-F  KatA1N-R  KatA1C-F  KatA1C-R  KatA2N-F  KatA2N-R  KatA2C-F  KatA2C-R  KatMnN-F  KatMnN-R  KatMnC-F  KatMnC-R  KatEN-F  KatEN-R  KatEC-F  KatEC-R  AhpCN-F  AhpCN-R  AhpFC-F  AhpFC-R  Gpx1N-F  Gpx1N-R  Gpx1C-F  Gpx1C-R  Gpx2N-F  Gpx2N-R  Gpx2C-F  Gpx2C-R  Gpx3N-F  Gpx3N-R  Gpx3C-F  Gpx3C-R  AhpCF-F  AhpCF-R  KatA1Q-F  KatA1Q-R  KatA2Q-F  KatA2Q-R  KatMnQ-F  KatMnQ-R  KatEQ-F  KatEQ-R  AhpCQ-F  AhpCQ-R  Gpx1Q-F  Gpx1Q-R  Gpx2Q-F  Gpx2Q-R  Gpx3Q-F  Gpx3Q-R  SmeXQ-F  SmeXQ-R  16S DNA-F  16S DNA-R | pRK415 with *ahpC* and *ahpF* genes; Tc^r^  pRK415 with a *P_gpx3_-xylE* promoter transcriptional fusion; Tc^r^  pRK415 with a *P_katA2_-xylE* promoter transcriptional fusion; Tc^r^  pRK415 with a *P_ahpC_-xylE* promoter transcriptional fusion; Tc^r^  5’- CGGAGCTCAGCAACGACGACA -3’  5’- TGATCTAGATCAGTGCGATCCA -3’  5’- GGTTCTAGAGATGGATGCGGAA -3’  5’- CAGGTCGACCAGCAGCAGGAT -3’  5’- CGCGTCGACGGGATGCTTCAT -3’  5’- GCTTCTAGAGCAGCCAGACGTCT -3’  5’- CCGTCTAGACGCCGACTTCTGGA -3’  5’- CGGAAGCTTGGGTGTCGCTGAT -3’  5’- CCGAGCTCGTCCAGGAGACGCA -3’  5’- CAATCTAGAGGCTGGCGAGCTG -3’  5’- GCATCTAGAACAGCGGCGAGCTA -3’  5’- GGGCATGCGGTGAGCCAGGAT -3’  5’- CTGGGTACCCAGGCTGTCCTTCT -3’  5’- GCTTCTAGAGTTCATCGCCGTTGC -3’  5’- CCTTCTAGATGAGGGTTCTGAT -3’  5’- CTGAAGCTTCTGTTTATCGGAGT -3’  5’- GCGGTACCAGCAGCTCGGTTT -3’  5’- CGTCTAGATCTGGGTGTT-3’  5’- CGTCTAGACCACCGAAGT-3’  5’- CCAAGCTTACCAGGTACTTCA-3’  5’- CCGGTACCGCGATTGCATCCT-3’  5’- GCATCTAGATCGAGAGCAGT-3’  5’- CCTCTAGACCAGCAAGGTGA-3’  5’- GCAAGCTTGGTGAACGACTT-3’  5’- GAGAGCTCGGCCTGCAGCTT-3’  5’- GATCTAGAAGGCAGTCGTCAT-3’  5’- GCTCTAGAGCGCCCGCATCAA-3’  5’- GGAAGCTTGACCCAGCTGAT-3’  5’- CAGAGCTCGATTCCCTACGCCGA-3’  5’- CCTCTAGAGTCGGCAAGCGAGGA-3’  5’- GATCTAGAGGTCATCGCCCGCT-3’  5’- CGAAGCTTGACCTGCGTGACACT-3  5’-GGAAGCTTCAGTGCCTGCTCAT-3’  5’-CATCTAGACAATGTTCCCGGTGATCT-3’  5’- TCCATTCCCGACCCGACCACC-3’  5’- GCTTGCGCGTTTCCGCATCC-3  5’- GTACCAGGACGTGGAGCAGT-3’  5’- CGTAGCTGTTCGGCTCGTAG-3  5’- TCCAACCCGCAGCTCGCCAG-3’  5’- GCCCCAGCAGCCGCATATCCTC-3  5’- GATGAGCGACCGCGCCATACC-3’  5’- CTGCACCGCCAGTTCCCATTCC-3  5’- CCGAGGTCTACATCGTCACC-3’  5’- AGGGTCTCGGAAACGTCAC-3  5’- CCAGCAAGCAGCAGGTCA-3’  5’- ACTTGACCCCGTAGGTGAGC-3  5’- CTGGTGGTGATCGGCTTC-3’  5’- GGCGATCGACCAGGAACT-3  5’- CTACCAGGGCAAGGTACTGC-3’  5’- GTACAGCGGGTGGGTGTC-3  5’-TACGACCGCCGCAAGCAACC-3’  5’- CAGCTCGAAGTAGTTGCGTGCC-3’  5’- GACCTTGCGCGATTGAATG -3’  5’- CGGATCGTCGCCTTGGT -3’ | This study  This study  This study  This study  This study  This study  This study  This study  This study  This study  This study  This study  This study  This study  This study  This study  This study  This study  This study  This study  This study  This study  This study  This study  This study  This study  This study  This study  This study  This study  This study  This study  This study  This study  This study  This study  This study  This study  This study  This study  This study  This study  This study  This study  This study  This study  This study  This study  This study  This study  This study  This study  This study  This study  6  6  6  6 |

**References**

1. Hu RM, Huang KJ, Wu LT, Hsiao YJ, Yang TC. Induction of L1 and L2 β-lactamases of *Stenotrophomonas maltophilia*. Antimicrob Agents Chemother. 2008;52:1198-200.

2. Wu CJ, Chiu TT, Lin YT, Huang YW, Li LH, Yang TC. Role of *smeU1VWU2X* operon in alleviation of oxidative stresses and occurrence of sulfamethoxazole-trimethoprim-resistant mutants in *Stenotrophomonas maltophilia*. Antimicrob Agents Chemother. 2018;62:e02114-17.

3. Simon R, O'Connell M, Labes M, Puhler A. Plasmid vector for the genetic analysis and manipulation of *Rhizobia* and other Gram-negative bacteria. Methods Enzymol. 1986;118:640-59.

4. Hoang TT, Karkhoff-Schweizer RR, Kutchma AJ, Schweizer HP. A broad-host-range Flp-FRT recombination system for site-specific excision of chromosomally-located DNA sequences: application for isolation of unmarked *Pseudomonas aeruginosa* mutants. Gene**.** 1998;212:77-86.

5. Keen NT, Tamaki S, Kobayashi D, Trollinger D. Improved broad-host-range plasmids for DNA cloning in gram-negative bacteria. Gene. 1988;70:191-197.

1. Chen CH, Huang CC, Chung TC, Hu RM, Huang YW, Yang TC. Contribution of resistance-nodulation-division efflux pump operon *smeU1-V-W-U2-X* to multidrug resistance of *Stenotrophomonas maltophilia*. Antimicrob Agents Chemother. 2011;55:5826-33.
