# Supplementary material for: Protection from hydrogen peroxide stress relies mainly on AhpCF and KatA2 in Stenotrophomonas maltophilia
Source: J Biomed Sci. 2020 Feb 25;27:37. doi: 10.1186/s12929-020-00631-4 (PMC7041247; doi:10.1186/s12929-020-00631-4)
Supplement: Supplementary file 2 — Additional file 2 Fig. S1. Analysis of putative OxyR binding motifs in the upstream regions of katA2 and ahpC genes. [file 12929_2020_631_MOESM2_ESM.docx]

**(A)**

*hypothetical protein*

***katA2***

**TGA**TCGGAGCGTGTCGGGATGCGAGCCGCCGGGCGACCGGAACAAGGCCCCGGCAATGAATTCCCGACAATCG**ATAGACAGAAGCTATAG**AAGTGAATGATTCATTCGATTATTCATTTCATCTGGCCATAGCTAAAGTCGAGTGAAGATGTTCCACCCCGTCTTCACCCGCAGGAGCCAGCCATGAGCCAGTCCGATAACAACAGCCCCAAGTGCCCGTATCACACCGCGCCGTCGCCTGCGGAAGGCGCCCAGCAGCAGCGTGAACTGAGCACCACGCCCAAGCAGAAGCACGGCGATGATCCGGTCACGCCG**ATG**

**(B)**

***ahpC***

***ahpF***

*Smlt0842*

**cat**tccgcaattgtagagccgtgcccatgctcggctgcttctggaacagcggaacatgggctcggttccagagatccgcagtcgagcgtggctcgacactacaaaaagctgcaatccgttcaggttctgccggagggaataaggaggggctatcggcccctttccgcgcctccgggagatttcccttgtcacacagccagttgcaggatgggagcggaaaggttcagtgcctgctcatgaccgaatg**atagactccatctatcaaatag**atgcaatcaatggattgttcttatcgccagcgagtcggtaacctagctcctgtcgattcacccactccccttcaccagaggaaaaacg**atg**

**Fig. S1. Analysis of putative OxyR binding motifs in the upstream regions of *katA2* and *ahpC* genes.** The orientation of gene is indicated by the arrow. The putative promoter region is underlined (promoter prediction: <https://fruitfly.org/seq_tools/promoter.html>). The putative OxyR binding motif is labelled in purple, based on the reported consensus sequence, ATAG-N10-ATAG, for OxyR of *E. coli*. (A) The DNA sequences upstream of the *katA2* gene and the putative OxyR binding motifs. (B) The DNA sequences upstream of the *ahpCF* gene and the putative OxyR binding motifs.
